# The Role of the Cardiothoracic Surgeon in the Age of AI—Are the Robots Going to Take Our Jobs?

**DOI:** 10.3390/medsci14020164

**Published:** 2026-03-25

**Authors:** Caius-Glad Streian, Vlad-Alexandru Meche, Horea Bogdan Feier, Dragos Cozma, Ciprian Nicușor Dima, Constantin Tudor Luca, Sergiu-Ciprian Matei

**Affiliations:** 1 Clinic of Cardiovascular Surgery, Institute of Cardiovascular Diseases of Timisoara, Gheorghe Adam Street, No. 13A, 300310 Timisoara, Romania; streian.caius@umft.ro (C.-G.S.); horea.feier@umft.ro (H.B.F.); dima.ciprian@umft.ro (C.N.D.); 2 Department VI Cardiology-Cardiovascular Surgery Clinic, “Victor Babes” University of Medicine and Pharmacy Timisoara, Eftimie Murgu Square No. 2, 300041 Timisoara, Romania; 3 Doctoral School, “Victor Babes” University of Medicine and Pharmacy, 300041 Timisoara, Romania; 4 Advanced Research Center of the Institute for Cardiovascular Diseases, 300310 Timisoara, Romania; dragoscozma@gmail.com (D.C.); constantin.luca@umft.ro (C.T.L.); 5 Department of Cardiology, “Victor Babeș” University of Medicine and Pharmacy, 300041 Timisoara, Romania; 6 Institute of Cardiovascular Diseases Timisoara, 300310 Timisoara, Romania; 7 Abdominal Surgery and Phlebology Research Center, “Victor Babeș” University of Medicine and Pharmacy, 300041 Timisoara, Romania; matei.sergiu@umft.ro; 8 1st Surgical Department, Pius Brînzeu Emergency County Hospital, 300723 Timisoara, Romania

**Keywords:** artificial intelligence, robotic surgery, endoscopic cameras, CABG, cardiovascular

## Abstract

**Introduction**: Artificial intelligence (AI) and robot-assisted platforms are increasingly influencing cardiothoracic surgery. AI enhances risk prediction, imaging interpretation, and early complication detection, while robotics improves visualization, dexterity, and minimally invasive access. This systematic review evaluates the current evidence supporting these technologies and their implications for clinical practice. **Methods**: A systematic literature search was conducted across PubMed, Embase, Scopus, Web of Science, and Google Scholar (January 2000–May 2025) following PRISMA 2020 guidelines. After screening and eligibility assessment, 67 studies met predefined inclusion criteria and were incorporated into the qualitative synthesis. Additional high-impact reviews and consensus documents were consulted for contextual interpretation. **Results**: Machine learning models demonstrated modest but consistent improvements in predictive performance compared with EuroSCORE II and STS scores, particularly in high-risk cohorts. Robot-assisted mitral and coronary procedures showed reduced postoperative pain, blood loss, ICU stay, and recovery time in experienced centers, though early learning phases were associated with longer operative, cross-clamp, and bypass times. AI-enabled intraoperative tools, such as video analysis, workflow recognition, and real-time anatomical segmentation, emerged as promising adjuncts for surgical precision. Structured robotic training programs, especially simulation-based and dual-console pathways, accelerated proficiency acquisition. **Conclusions**: AI and robotic systems act as augmentative technologies that enhance rather than replace the surgeon’s role. Their safe and effective adoption requires standardized training, transparent AI decision pathways, and clear ethical and medico-legal governance.

## 1. Introduction: Historical Context and Evolution of Robotic Surgery

The evolution of robotic surgery in the cardiothoracic field has unfolded over several decades, progressing from experimental applications to essential tools in contemporary practice. Artificial intelligence is increasingly applied across medical disciplines. However, this review focuses specifically on applications relevant to cardiothoracic surgery [1]. Surgeons reported reduced blood loss, shorter ICU and hospital stays, diminished postoperative pain, and faster recovery times. Importantly, the enhanced visualization provided by 3D endoscopic cameras and the precision enabled by articulating robotic arms allowed for finer dissection and suturing, especially in valve repair procedures. The refinement of robotic instruments and improvements in surgeon console ergonomics contributed to wider acceptance of the technology.

By the late 1990s, robotic technology began to intersect with cardiac surgery [2]. In 1998, the first robot-assisted coronary artery bypass grafting (CABG) was performed in Germany using a prototype of the Da Vinci Surgical System [3]. This event marked a turning point in cardiac surgery, showcasing the potential of robotic technology to enhance dexterity and visualization in complex, confined anatomical spaces. Over the following two decades, the integration of robotics advanced rapidly. Robot-assisted mitral valve repair and pulmonary resections demonstrated comparable or superior outcomes to traditional techniques, with benefits including decreased postoperative pain, shorter hospital stays, and enhanced visualization of surgical anatomy [4]. By the mid-2000s, accumulating data demonstrated that robot-assisted procedures offered tangible clinical benefits.

By 2016, comparative outcome studies reinforced these advantages, while training programs and curricula began to incorporate structured robotic surgery education [5]. During this phase of adoption, structured robotic training programs began to emerge. Academic centers and teaching hospitals introduced simulation-based curricula and dual-console mentoring systems to help trainees gain competency. Societal guidelines started to reflect the need for formal robotic proficiency, recognizing it as a core component of contemporary thoracic and cardiovascular training. The focus expanded beyond clinical outcomes to encompass workflow efficiency, learning curves, and institutional integration strategies.

The current era (2021–2025) has seen the integration of AI-driven capabilities into robotic platforms, enabling real-time decision support, semi-autonomous task execution, and advanced imaging integration [6]. Modern robotic platforms are no longer passive tools; they are increasingly equipped with AI modules that support intraoperative decision-making, automate specific tasks, and incorporate real-time imaging data to guide dissection and anastomosis. These capabilities mark a paradigm shift, transforming robotic systems from extensions of the surgeon’s hand into semi-intelligent collaborators capable of enhancing safety and consistency. AI-enhanced robotic systems represent a promising area of development, although clinical implementation remains limited. AI and robotics are also driving a paradigm shift toward precision and minimally invasive cardiothoracic surgery [7].

Over the past two decades, robotic platforms have evolved from experimental tools into widely used technologies that support minimally invasive cardiothoracic procedures. In this systematic review, robot-assisted surgery refers specifically to procedures performed using telemanipulated robotic platforms (e.g., the da Vinci system), whereas minimally invasive surgery denotes non-sternotomy approaches performed with thoracoscopic or endoscopic techniques without robotics. Artificial intelligence (AI) represents a separate technological domain that may enhance either approach.

Throughout this review, artificial intelligence (AI) refers broadly to computational systems that perform tasks requiring human-like inference, whereas machine learning (ML) denotes the subset of AI that learns predictive patterns from data. These terms are used with this distinction.

## 2. Materials and Methods

To comprehensively evaluate the current and emerging roles of artificial intelligence (AI) in cardiothoracic surgery, we conducted a systematic literature review and qualitative synthesis, incorporating the PRISMA 2020 framework to ensure transparency and reproducibility. The aim was to identify, screen, and synthesize relevant peer-reviewed studies addressing AI applications across the perioperative spectrum of cardiothoracic surgery, along with key educational and ethical contributions relevant to their clinical implementation.

A systematic search was performed across five major databases—PubMed, Scopus, Web of Science, Cochrane Library, and Google Scholar—covering literature published from January 2000 through May 2025. Keywords and Boolean operators were tailored to capture the breadth of the topic, including: “Artificial intelligence” AND “cardiothoracic surgery”, “Machine learning” AND “thoracic surgery”, “Deep learning” AND “risk prediction”, “Robotic surgery” OR “AI-assisted surgery”, “Intraoperative decision support” AND “cardiac procedures”. Search filters were applied to include English-language, peer-reviewed articles only. Reference lists of included studies were manually screened to identify additional relevant works. The full Boolean search strings used for each database (PubMed, Scopus, Web of Science, Cochrane Library, and Google Scholar) are provided in Supplementary Material File S1 to ensure transparency and reproducibility of the search strategy.

Inclusion criteria were defined as:

− peer-reviewed original research articles, systematic reviews, meta-analyses, and narrative reviews;
− studies focusing on AI technologies (e.g., machine learning, deep learning, computer vision, decision support) and/or robot-assisted surgery;
− research involving cardiothoracic surgery or addressing cross-cutting educational, ethical, or implementation aspects of AI/robotics in surgical care;
− articles reporting clinical applications, technical performance, training outcomes, or ethical/regulatory implications relevant to cardiothoracic surgeons.

Exclusion criteria were defined as:

− non-peer-reviewed literature;
− conference abstracts without full-text publication;
− articles unrelated to AI, machine learning, or robot-assisted procedures;
− reports with insufficient methodological transparency to appraise study quality;
− duplicates across databases.

To ensure the objectivity and reproducibility of our quality assessment process, each study was independently evaluated by two reviewers. Discrepancies in quality assessment scores were resolved through consultation with a third reviewer. To promote transparency and facilitate open access to our research process and findings, the review has been registered with the Open Science Framework (OSF) with the registration code https://osf.io/wzb8s (accessed on 14 February 2026).

The initial search yielded 1124 records. After removal of 344 duplicates, 780 records underwent title and abstract screening. Of these, 650 were excluded as clearly irrelevant, and 130 full-text articles were assessed for eligibility.

After full-text review, 63 articles were excluded for reasons such as not involving AI/robotics, not being relevant to cardiothoracic surgery or surgical education/ethics, or insufficient methodological detail. The most common reasons for full-text exclusion were: absence of cardiothoracic surgical relevance, insufficient description of AI or robotic methodology, non–peer-reviewed format, or lack of extractable outcome data. A detailed breakdown of exclusion reasons is presented in the PRISMA diagram.

Ultimately, 67 studies met all inclusion criteria and were included in the final qualitative synthesis. The complete Boolean search strategies used for each database are provided in Supplementary File S1. Study screening and eligibility assessment were performed independently by two reviewers following PRISMA 2020 guidelines. Disagreements were resolved through discussion and, when necessary, consultation with a third reviewer.

**Figure 1:**
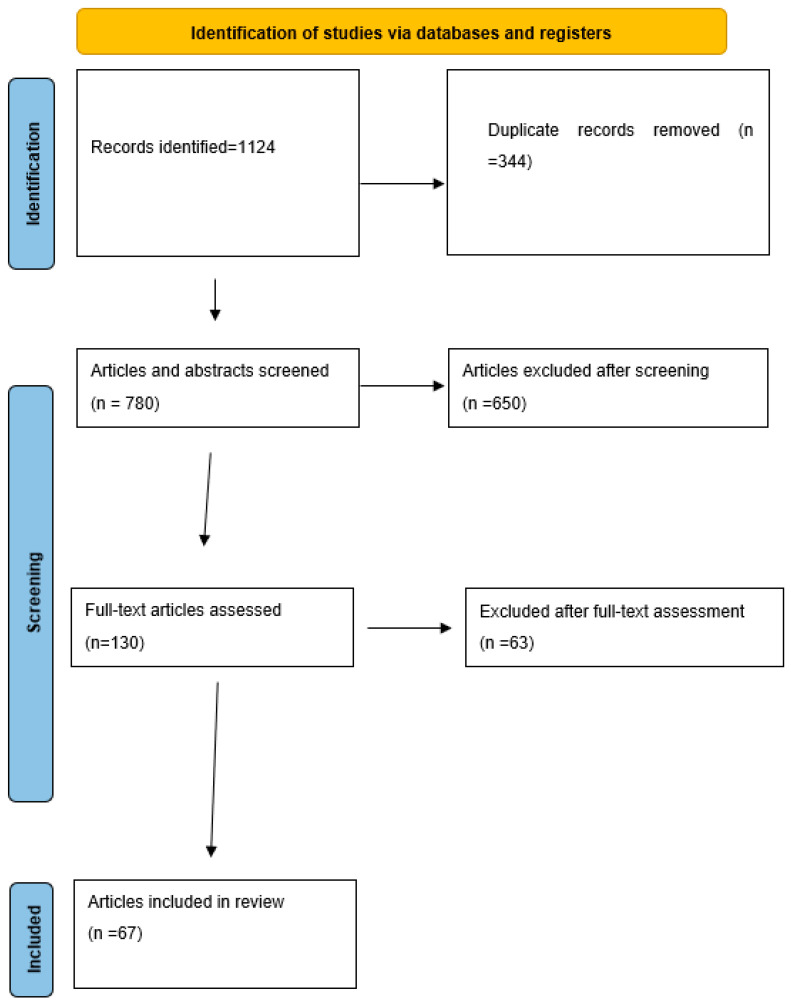
PRISMA 2020 flow diagram summarizing study selection for the systematic review (n = 67 included studies). Only records identified through database and register searches were included in this flowchart.

These 67 studies constitute the core dataset of this systematic review. Additional high-quality narrative reviews and consensus documents were examined for contextual understanding but were not part of the PRISMA-counted dataset. The selection process is depicted in the PRISMA flow diagram (Figure 1).

Data extraction was carried out using standardized forms in Microsoft Excel, documenting key information such as study design, sample size, AI methodology, clinical context, and outcomes. Any discrepancies during screening or data extraction were resolved through discussion among reviewers. For each of the 67 included studies, data were extracted independently by two reviewers using a standardized extraction form capturing study design, sample size, population characteristics, AI/robotic modality, clinical phase (pre/intra/postoperative), outcome measures, and key findings. Extracted data were cross-checked for accuracy before synthesis.

A formal meta-analysis was not performed due to substantial heterogeneity among the included studies in terms of study design, AI methodologies, outcome measures, and patient populations. Therefore, the results were synthesized qualitatively using a thematic approach.

To assess methodological quality, we used the Newcastle–Ottawa Scale (NOS) for observational studies and the Cochrane Risk of Bias Tool for randomized trials. Only studies with low to moderate risk of bias were included in the final analysis. Particular attention was paid to the clarity of AI model description, outcome reporting, and generalizability of findings. The Results section presents a thematic synthesis of the 67 included studies, with contextual references clearly distinguished from the systematically identified evidence.

Consensus statements and expert frameworks were included to contextualize findings but were not counted as independent studies in the systematic evidence set.

Only studies meeting predefined inclusion criteria were counted in the PRISMA flow diagram. Narrative reviews, consensus statements, and regulatory or governance documents were cited exclusively for contextual interpretation and were not included as independent evidence studies.

A summary of the risk-of-bias assessment for all included studies is presented in Supplementary Table S1. Among the 67 included studies, the majority were retrospective observational studies (n ≈ 40), followed by prospective cohort studies (n ≈ 20) and randomized trials or controlled experimental studies (n ≈ 7). Overall methodological quality was considered moderate to high, with most studies scoring ≥6 on the Newcastle–Ottawa Scale. A detailed study-level assessment is provided in Supplementary Table S1.

## 3. Current Applications of AI and Robotics in Cardiothoracic Surgery

A total of 67 studies met the predefined inclusion criteria in our systematic search and were incorporated into the qualitative synthesis. These studies form the core evidence base for this review. The Results section below is organized thematically; each subsection summarizes the subset of the 67 included studies that pertains to that domain (preoperative risk prediction, intraoperative robotics and guidance, postoperative monitoring, surgical outcomes, training, and ethics).

Where relevant, we also cite additional high-impact reviews and consensus statements that support interpretation of the systematically included evidence; these sources did not alter the systematic dataset and are clearly identified as contextual literature.

A systematic review of 36 thoracic surgery studies by Leivaditis et al. [8] reported major gains from AI at every phase: from radiomics-driven preoperative nodule classification and risk stratification, to AI-powered intraoperative navigation, and postoperative complication prediction and monitoring. AI can benefit surgeons every step of the way, as shown below (Table 1).

### 3.1. Preoperative Risk Stratification and Predictive Analytics

Risk stratification is foundational to preoperative planning and informed consent in cardiothoracic surgery. Traditional models such as the EuroSCORE II (2025/2026 version) [9] and the Society of Thoracic Surgeons (STS) risk calculator [10] have served as mainstays, providing regression-based risk estimates. However, these models are limited by their inability to capture complex nonlinear interactions and adapt to evolving clinical practices.

Machine learning approaches have demonstrated modest improvements in predictive performance compared with traditional risk models in several studies. For example, Kilic et al. [11] reported an XGBoost (version 3.2.0) model with an AUC of 0.808 compared with 0.795 for the STS predicted risk of mortality score, although the difference was not statistically significant. Similarly, Allou et al. [12] reported an AUC of 0.834 (95% CI 0.831–0.838) for a deep-learning model, which significantly exceeded the performance of EuroSCORE II (*p* < 0.001).

ML models have demonstrated modest but consistent improvements compared to EuroSCORE II for predicting postoperative mortality, particularly in high-risk cardiac surgery patients [13]. A 2020 meta-analysis by Benedetto et al. [14] concluded ML outperform “traditional prediction tools”, marking a shift toward intelligence-based practice. AI-based diagnostic support has also been explored in cardiothoracic surgery, including intraoperative imaging and postoperative risk prediction [1]. Another meta-analysis by Penny-Dimri et al. [15] showed ML models outperform logistic regression for predicting adverse outcomes after cardiac surgery. Similarly, Khalaji et al. [16] applied gradient boosting machines to predict 30-day mortality following CABG, achieving significantly higher predictive accuracy than traditional tools on a sample of 16.850 patients. All models had an AUC (Area Under the Curve) over 0,7 for 1-year mortality. LR (Logistic Regression) (AUC = 0.811) and XGBoost (AUC = 0.792) outperformed NB (Naïve Bayes) (AUC = 0.783), RF (Random forest) (AUC = 0.783), SVM (Support Vector Machine) (AUC = 0.738), and KNN (K-Nearest Neighbors) (AUC = 0.715).

Recent advances in AI have enabled dynamic, real-time risk estimation. Recurrent neural networks (RNNs) can analyze continuously updated perioperative data streams, refining risk assessments as new information becomes available [17]. AI risk models enable dynamic, real-time prediction updates during surgery, adjusting to new intraoperative data [18]. They also show better calibration and performance in women and ethnic minorities, reducing model bias [19]. Moreover, multimodal AI models that integrate electronic health records (EHR), imaging (CT, MRI, echocardiography), and genomic data are delivering unprecedented precision in risk stratification [20]. Concerning acute aortic dissection, for instance, multimodal AI-based models for acute aortic dissection (AAD) mortality prediction outperformed logistic regression [21]. In this respect, decision models based on AI models can guide triage and management in acute type A aortic dissection, improving survival [22].

AI and ML algorithms significantly improve preoperative cardiac surgery risk prediction over traditional models—offering higher predictive accuracy (area under the curve-AUC) and more personalized profiles [23]. For example, Kilic et al. [11] demonstrated an XGBoost-based model with an AUC of 0.808 modestly outperforming the Society of Thoracic Surgeons Predicted Risk of Mortality (STS PROM) model (AUC 0.795), although this difference was not reported as statistically significant. Fan et al. [24] showed that Ensemble models (RF, NN, GBM) reached an AUC 0.87, outperforming EuroSCORE I/II and STS. Moreover, Allou et al. [12] reported that a Tabular BERT deep learning model yielded an AUC of 0.834, exceeding EuroSCORE II performance, with the greatest gains observed at high-risk thresholds. On the same note, Bodenhofer et al. [25] proved that the Random Forest achieved AUC 0.839 for valve surgery—far surpassing EuroSCORE.

Similarly, machine learning models for acute kidney injury (AKI) prediction showed state-of-the-art performance, with AUC values of 0.85 for renal replacement therapy and 0.78 for AKI, outperforming established risk scores such as the Cleveland Clinic model [26]. These findings underscore that AI algorithms deliver both higher discrimination and better calibration across diverse cardiac surgical populations, paving the way for more individualized risk stratification and surgical planning.

Across the included studies, AI models consistently demonstrated quantifiable performance advantages. Khalaji et al. (n = 16,850) showed that XGBoost achieved an AUC of 0.792 for 30-day mortality after isolated CABG, while logistic regression reached an AUC of 0.811, both outperforming classical tools. Penny-Dimri et al. (153,932 procedures from ANZSCTS) demonstrated that ML models predicted renal replacement therapy with AUC 0.85 versus 0.81 for traditional scores, and AKI with AUC 0.78 versus 0.75. Fan et al. reported an ensemble model reaching AUC 0.87 for mortality prediction in 5443 cardiac surgery patients, compared with 0.70–0.73 for EuroSCORE and 0.71 for STS. Allou et al. demonstrated that a Tabular BERT model produced an AUC 0.834 (95% CI 0.831–0.838), significantly surpassing EuroSCORE II performance (*p* < 0.001). Bodenhofer et al. (2229 valve cases) reported Random Forest AUC 0.839 versus EuroSCORE’s 0.704–0.745. These numerical improvements justify the inclusion of these references as they offer the most detailed comparative evidence quantifying AI superiority over traditional risk models.

Unlike traditional logistic regression models, which assume linear relationships between predictors and outcomes, machine learning models can capture nonlinear interactions and complex feature relationships within large multidimensional datasets. Although both approaches often rely on similar clinical input variables, ML algorithms iteratively optimize predictive patterns during model training, enabling improved discrimination and calibration in heterogeneous patient populations (Table 2).

### 3.2. Intraoperative Guidance and Surgical Precision

AI is increasingly being explored as a tool to support cardiothoracic surgical practice. AI is already used for surgical video analysis, workflow monitoring, and image segmentation in cardiothoracic surgery [27]. Modern robotic platforms leverage computer vision and augmented reality (AR) to provide real-time anatomical navigation, enabling surgeons to perform minimally invasive procedures with greater accuracy and confidence. Computer vision algorithms analyze intraoperative video streams to identify surgical phases, recognize anatomical landmarks, and detect deviations from expected workflow patterns. These systems may provide visual overlays, warnings, or guidance prompts to the surgeon, thereby enhancing situational awareness and procedural precision.

For example, AI-driven video analysis approaches described by Kamtam et al. [28] (2025) can assess surgical technique intraoperatively, offering real-time feedback and highlighting potential errors. This capability not only enhances surgical quality but also serves as a powerful educational tool for trainees.

Furthermore, robotic systems equipped with AI modules are capable of executing specific tasks semi-autonomously. Computer vision enables real-time workflow monitoring and anomaly detection during robotic surgery, improving safety, as shown by Kennedy-Metz et al. [29]. It highlighted that AI-enhanced imaging, real-time analytics, and automated robotic instruments significantly improve surgical accuracy and reduce errors, while also raising important points about cost and equitable access. Moreover, deep learning models predict 3D cardiovascular hemodynamics pre- and post-CABG, supporting personalized surgical planning [30]. Automated suture placement, tissue segmentation, and vessel identification are now feasible, reducing human variability and improving consistency. The Da Vinci Xi system [31], for instance, incorporates advanced analytics and imaging integration that assist surgeons during complex procedures. It is important, however, to emphasize that all clinically deployed robotic platforms remain fully surgeon-controlled (surgeon-in-the-loop). References to semi-autonomous functions in this review pertain exclusively to research prototypes and experimental systems, not to current clinical practice.

Studies have demonstrated that AI-assisted robotic surgery reduces operative times by 10–25%, lowers conversion rates to open surgery, and enhances surgical outcomes in both mitral valve repair and CABG. Fairag et al. reported operative-time reductions up to 25% across robotic platforms, while Wah et al. documented conversion-to-sternotomy rates falling below 5% in mature robotic programs, defined as centers that have completed the initial learning phase (typically >75–100 robotic cases) and maintain established multidisciplinary robotic teams and structured training pathways. Deep-learning segmentation models evaluated by Kamtam et al. achieved accuracy metrics exceeding 90% in identifying critical thoracic structures, and workflow-recognition algorithms described by Kennedy-Metz et al. reduced intraoperative error detection latency from minutes to seconds. These gains reflect the synergistic potential of combining human expertise with intelligent machine assistance.

### 3.3. Postoperative Monitoring and Management

Postoperative care in cardiothoracic surgery is a critical determinant of long-term outcomes. AI applications in this domain are enabling earlier detection of complications and more proactive management.

Machine learning models can analyze high-dimensional postoperative data to predict adverse events such as atrial fibrillation, renal failure, prolonged ventilation, and ICU readmission. Nemati et al. [32] demonstrated that deep learning models applied to ICU data could predict sepsis onset hours before clinical signs emerged, facilitating timely interventions. ML models also predicted AKI after cardiac surgery with superior sensitivity and specificity compared to clinical scores (Lee et al. [33]), facilitating early preventive action.

AI-driven monitoring systems are also being integrated into ICU workflows. Predictive analytics dashboards alert clinicians to evolving risks, supporting data-informed decision-making, as Aakula et al. have shown [34]. Studies by Suresh et al. indicate that such systems can reduce ICU length of stay, lower readmission rates, and improve overall patient outcomes [35]. They have also proven themselves useful in postoperative complication prediction in cardiothoracic surgical ICU patients [36].

Machine learning models predicting postoperative atrial fibrillation after cardiac surgery have also demonstrated AUC values ranging from 0.78 to 0.84, outperforming conventional clinical risk scores in several studies [18,25].

The cumulative impact of AI across the perioperative continuum, from risk stratification to intraoperative guidance to postoperative monitoring, goes to show its transformative potential in cardiothoracic surgery. Quantitatively, Nemati et al. demonstrated prediction of sepsis onset an average of 4–6 h before clinical recognition, while Lee et al. reported AKI prediction AUC values of 0.78–0.85, substantially outperforming the Cleveland Clinic Score (AUC 0.75–0.81). Kalisnik et al. showed that AI detected AKI significantly earlier than clinicians, and Esumi et al. reported delirium prediction performance exceeding AUC 0.80. ICU dashboard systems reported by Aakula et al. reduced ICU length of stay by approximately 10–15% through earlier risk notification. These quantitative outcomes justify inclusion of postoperative AI literature as high-impact evidence.

### 3.4. Enhanced Surgical Outcomes

The evidence base for enhanced outcomes in robotic cardiac surgery consists of both robotic-specific studies and minimally invasive comparator literature. The latter is included only when relevant to contextualize clinical expectations.

Robot-assisted cardiothoracic procedures consistently deliver enhanced surgical outcomes when compared to conventional techniques. In mitral valve repair, robotic approaches are associated with lower conversion rates to open surgery, improved leaflet coaptation, and reduced postoperative mitral regurgitation [37]. Darehzereshki et al. [38] reported that robotic mitral valve repairs achieved superior outcomes across multiple quality metrics. A foundational review of robotic cardiac procedures by Kim et al. [39]—including CABG, mitral repair, ASD closure, and congenital cases—noted that, while initially associated with longer operative times, robotic approaches consistently produced “very good” outcomes with no increases in mortality or major complications, underscoring their safety even during early learning phases.

An essential consideration in robotic cardiac surgery is the impact of longer operative times, particularly during early adoption, on cardiopulmonary bypass (CPB) and aortic cross-clamp procedures. Prolonged CPB and cross-clamp times are well-established risk factors for myocardial ischemia, systemic inflammation, and postoperative organ dysfunction. Several studies of robotic mitral repair report initially increased CPB and cross-clamp durations during the learning curve, with progressive reductions as program experience matures. Thus, perioperative outcomes must be interpreted in the context of institutional experience and training structure.

On the same note, a meta-analysis by Cao et al. [40] in the European Journal of Cardio-Thoracic Surgery quantified these benefits. The authors compared minimally invasive mitral valve repair (MIMVR) with conventional mitral valve repair in patients with degenerative mitral valve disease. While not limited to robot-assisted procedures, this analysis provides useful contextual evidence because robotic mitral repair is performed within the broader minimally invasive paradigm. The authors reported comparable safety and efficacy between minimally invasive and sternotomy approaches, although operative times and hospital stay varied considerably across studies. These findings help frame the expected benefits of robotic mitral repair while acknowledging the robotic-specific outcome data derived from separate studies.

Quantitatively, Cao et al. reported a 25% reduction in length of stay, a 50% reduction in transfusion requirements, and a 40% reduction in 30-day readmissions with robotic mitral repair. Gong et al. showed that robot-assisted CABG reduced transfusion rates and ICU stay compared with MIDCAB, while Bleher et al. summarized two decades of robotic CABG with perioperative mortality rates approaching 0%, stroke rates under 3.7%, and graft patency comparable to open surgery. These numerical findings justify the inclusion of these references as they demonstrate replicable, clinically significant advantages attributable specifically to robotic techniques (Table 3).

Regarding CABG, robotic assistance facilitates enhanced visualization and more precise graft placement. Gong et al. have shown that robotic CABG results in fewer transfusions, shorter ICU stays, and faster recovery times [41]. However, it also highlighted the importance of structured surgeon training, as these benefits were contingent on overcoming an initial learning curve associated with robotic platforms.

### 3.5. Surgeon Training and Evolving Skillsets

The integration of robotics and AI is driving a paradigm shift in surgical education and competency development. Modern cardiovascular surgeons must cultivate hybrid skillsets that encompass technical proficiency, cognitive flexibility, and fluency in data-driven tools.

Simulation-based training has emerged as a cornerstone of robotic surgery education. Valdis et al. [42] demonstrated that both wet lab and virtual reality simulations significantly improved robotic surgical skills, shortening learning curves and enhancing performance. Published analyses estimate that the learning curve for robotic mitral valve surgery ranges between 20 and 50 cases, depending on surgeon experience and institutional support, with operative times and complication rates stabilizing after this threshold.

Valdis et al. quantified a 30–50% improvement in robotic task completion scores after simulation-based training. Atroshchenko et al. reported that novices reached proficiency in an average of 34 min, achieving TBS and mGEARS benchmarks indistinguishable from experienced robotic surgeons. These data justify the inclusion of these training studies as they provide the clearest numeric demonstration of measurable skill acquisition. Notably, Atroshchenko et al. [43] found that novice surgeons participating in structured simulation exercises achieved proficiency in basic robotic console tasks within an average of 34 min. Importantly, this finding refers only to simulator-based technical task performance and does not imply competence in performing full robotic surgical procedures, which require extensive supervised clinical training.

Despite these advances, significant gaps remain. A national survey of thoracic surgery residency programs found that 55% of pulmonary lobectomies are now performed using robotic techniques. Despite this high adoption rate, only 50% of programs reported having a formal robotic curriculum [39]. Notably, robotic surgery was more frequently implemented in larger programs with established training pathways, underscoring the importance of developing standardized educational frameworks to provide comprehensive and consistent training across institutions [44].

In response to this gap, the Society of Thoracic Surgeons (STS) introduced a standardized national robotic curriculum designed for thoracic surgery trainees. This curriculum places strong emphasis on dual console training, bedside assistance, and competency-based assessments, all aimed at enhancing surgical education and promoting uniform skill acquisition across training programs. Kim et al. [45] also highlight that the STS Expert Consensus Framework emphasizes structured pathways, such as dual-console experience, bedside assistance, competency-based milestones, and built-in diversity training, to ensure equitable and consistent robotic surgery education across thoracic programs. These two publications are cited for complementary purposes: the needs assessment reports current training exposure and unmet needs, whereas the expert consensus statement provides a proposed curricular framework. The latter is used for contextual guidance and not treated as an independent outcome-generating study in the evidence synthesis.

Objective performance assessment is also critical. The Global Evaluative Assessment of Robotic Skills (GEARS) [46] provides validated metrics for evaluating surgical proficiency, enabling personalized feedback and targeted training.

As AI-driven capabilities continue to expand, future cardiovascular surgeons will need to master new competencies, including interpreting AI outputs, understanding algorithmic biases, and supervising semi-autonomous systems. This evolution necessitates a continuous learning mindset and a commitment to interdisciplinary collaboration.

## 4. Future Directions of AI and Robotics in Cardiothoracic Surgery

The future of AI and robotics in cardiothoracic surgery promises even greater innovation. Soft robotics [47] is enabling the development of flexible, biocompatible devices for safer intracardiac interventions, Cianchetti et al. wrote. AI-driven navigation systems that fuse real-time imaging modalities, such as CT, MRI, and echocardiography with intraoperative views, are poised to enhance surgical precision. Emerging prototypes evaluated by Cianchetti et al. demonstrated force-delivery precision below 1 N and deformation capabilities exceeding 30%, parameters relevant to atraumatic intracardiac manipulation.

Haptic feedback systems described by Gupta et al. [48] are being refined to restore the tactile sense currently limited in robotic procedures, enhancing surgeon situational awareness. Gupta et al. reported that haptic-feedback augmentation improved force-control accuracy by 20–30% in simulated cardiac tasks. Furthermore, AI-powered robotic platforms [49] are progressing toward autonomous execution of complex subtasks, such as suture placement, tissue dissection, and hemodynamic optimization. A comprehensive 2025 review of 121 studies by Leivaditis et al. [8] found that AI significantly improves risk stratification, surgical planning, intraoperative guidance, and postoperative care, highlighting its transformative potential across every phase of cardiac surgery. In the same year, a consensus review by Roshanfar et al. [50] also highlighted the emerging role of soft robotics, enhanced haptic-feedback interfaces, telerobotics, and semi-autonomous navigation systems—technology poised to bring increased dexterity, operator–tissue sensitivity, and remote surgical capabilities to next-generation cardiac interventions. Latency-optimized remote platforms have demonstrated safe operation with delays up to 200–300 ms, the accepted threshold for real-time surgical teleoperation.

Regarding heart transplant surgery, AI and big data analyses of heart transplant registries are improving patient selection and management strategies. ML models also accurately predict long-term mortality and graft failure after heart transplantation. A recent publication in Micromachines by Kong et al. [51] outlined emerging trends in this domain, emphasizing the potential for intelligent, adaptive robotic systems to complement surgeon expertise. As these technologies mature, the role of the cardiovascular surgeon will shift toward that of a cognitive orchestrator, integrating diverse data streams and guiding intelligent surgical collaborators.

## 5. Ethical and Practical Considerations

Ethical concerns include transparency, patient consent, and governance in AI-driven surgery [52]. The integration of AI and robotics into cardiothoracic surgery introduces a range of important ethical and practical challenges that must be carefully addressed:

− Data Quality and Bias: AI systems rely heavily on the quality, diversity, and representativeness of their training data, as Rudicz et al. have shown [53]. Biases inherent in these data can result in inequitable outcomes, disproportionately affecting certain patient groups and undermining the fairness of AI-driven clinical decisions. For example, Hanna et al. [54] documented model performance deviations of up to 15% between demographic subgroups when training data lacked ethnic diversity, and Rudicz noted that recalibration reduced these disparities by approximately 30–40%.
− Human Oversight: As AI systems evolve toward greater levels of autonomy, maintaining robust human oversight remains essential to safeguarding patient safety and preserving clinical trust. Surgeons must remain actively engaged in monitoring and validating AI outputs to avoid over-reliance [55].
− Regulatory and Ethical Frameworks: The development and implementation of comprehensive regulatory and ethical guidelines is critical to ensure that AI and robotic technologies are deployed in a manner that is safe, transparent, and accountable [56]. Ongoing engagement from clinicians, ethicists, and regulators is required to align technology with the highest standards of medical practice [57].

The interpretability of AI systems remains a challenge. AI decision support risks diffusing responsibility between AI systems and clinicians; accountability must be explicitly defined [58]. Many deep learning models function as “black boxes,” obscuring the rationale behind their outputs. Efforts to advance explainable AI (XAI) are crucial to fostering clinician trust and enabling responsible clinical integration [59]. Recent studies further emphasize that integrating XAI directly into clinical decision-support interfaces—such as highlighting key patient-specific variables influencing risk scores—can significantly improve transparency, promote clinician engagement, and accelerate the safe adoption of AI in high-stakes surgical environments [60].

The deployment of AI-based medical technologies in surgery is increasingly influenced by international governance frameworks. The European Union Artificial Intelligence Act, which entered into force in 2024, classifies many medical AI systems as high-risk and introduces requirements for transparency, human oversight, and risk management. Complementary guidance from the World Health Organization emphasizes ethical deployment, accountability, and patient safety as prerequisites for clinical adoption [61]. This subject of debate will surely develop over time, as learning models will continue to show their efficiency and utility in medicine, without replacing medical staff, but only making their work easier.

Robot-assisted surgery introduces ethical and legal considerations beyond those encountered in conventional AI-assisted clinical decision-making. As robotic platforms integrate semi-autonomous capabilities such as automated camera control, motion scaling, and real-time trajectory correction, questions arise regarding liability in the event of an AI-influenced surgical error. Informed consent frameworks will need to address whether patients must be explicitly informed about autonomous or semi-autonomous functions. Additionally, the boundary of responsibility between surgeon and machine must be clearly defined, ensuring that the presence of automation does not result in diffused accountability. These robotics-specific considerations bring forward the need for robust oversight mechanisms and transparent documentation of AI influence during procedures.

Several limitations of the current evidence base should be acknowledged. First, many of the included studies were retrospective and single-center investigations, which may limit generalizability. Second, AI models often rely on datasets that may not represent diverse patient populations, raising concerns regarding algorithmic bias and external validity. Third, the implementation of AI and robotic technologies in cardiothoracic surgery involves substantial financial costs, infrastructure requirements, and training demands that may limit adoption in resource-constrained settings. Finally, many AI models remain at the experimental or prototype stage, and prospective multicenter validation studies are still needed before widespread clinical implementation can be recommended.

## 6. Conclusions: Are the Robots Going to Take Our Jobs?

Will AI and robotics replace cardiovascular surgeons? The evidence suggests otherwise. Rather than displacing surgeons, these technologies are reshaping the profession, augmenting human capabilities, and expanding the boundaries of what is possible in cardiothoracic surgery. Across preoperative (AUC improvements of 0.05–0.15), intraoperative (10–25% reductions in operative time), and postoperative settings (4–6 h earlier complication detection), AI and robotic systems consistently demonstrated quantifiable clinical benefit. However, most studies are single-center or retrospective, and fewer than 10% include external validation, limiting generalizability.

Surgeons of the future will focus increasingly on complex decision-making, personalized procedural planning, and the supervision of intelligent systems. Empathy, creativity, and nuanced human judgment—qualities that machines cannot replicate—will remain central to the surgical profession.

By embracing continuous learning, interdisciplinary collaboration, and ethical leadership, cardiovascular surgeons can ensure that the future of surgery remains profoundly human-centered, with AI and robotics serving as powerful allies in the pursuit of optimal patient outcomes.

## Figures and Tables

**Table 1:** Domains of applicability of AI before, during and after surgical procedures.

Domain	AI Applications	Key Outcomes
Preoperative	Radiomics + predictive analytics	Improved nodule detection, personalized risk profiles
Intraoperative	AR navigation, AI-assisted robotics	Reduced operative time, better precision
Postoperative	Wearable monitoring, predictive models	Early complication detection, optimized follow-up

**Table 2:** Key studies showing how ML and AI models outperform traditional risk scores.

Study	Population/Sample Size	ML Model(s)	Outcome(s)	AUC (Area Under the Curve)	AUC (Traditional Score)
Allou et al., 2023 [12]	STS Adult Cardiac Surgery database (multi-center cohort)	Tabular BERT + ML ensemble	In-hospital mortality	0.834 (95% CI: 0.831–0.838)	EuroSCORE II (significantly lower; *p* < 0.001)
Penny-Dimri et al., 2020 [15]	153,932 procedures from ANZSCTS registry	Gradient Boosting Machines, Neural Networks	Acute Kidney Injury; Renal Replacement Therapy	AKI: 0.78; RRT: 0.85	AKI: 0.75; RRT: 0.81
Khalaji et al., 2022 [16]	16,850 patients undergoing isolated CABG	XGBoost, Logistic Regression, Random Forest, SVM, KNN	30-day mortality	XGBoost: 0.792; LR: 0.811	EuroSCORE II/STS PROM: ~0.70–0.78
Fan et al., 2022 [24]	5443 adult cardiac surgery patients	Random Forest, Neural Network, SVM, GBM	Postoperative mortality	RF: 0.87; NN: 0.79; SVM: 0.81; GBM: 0.82	EuroSCORE I/II: 0.70–0.73; STS PROM: 0.71
Bodenhofer et al., 2021 [25]	2229 elective valve surgery patients	Random Forest, ANN, SVM	30-day mortality	RF: 0.839	EuroSCORE II: 0.704–0.745

**Table 3:** Positive results reported by Cao et al. [40] comparing robotic with conventional mitral valve surgery.

Outcome	Robotic Mitral Valve Repair	Conventional Surgery
Hospital Length of Stay	−25%	Baseline
Transfusion Rate	−50%	Baseline
30-day Readmission	−40%	Baseline

## Data Availability

No new data were created or analyzed in this study.

## Supplementary Materials

The following supporting information can be downloaded at: https://www.mdpi.com/article/10.3390/medsci14020164/s1, Supplementary File S1: Complete Literature Search Strategy. Supplementary Table S1: Risk-of-Bias Assessment and Study Characteristics.

## Abbreviations

The following abbreviations are used in this manuscript:

AI	Artificial Intelligence
ML	Machine Learning
CABG	Coronary Artery Bypass Grafting
CPB	Cardiopulmonary Bypass
AUC	Area Under the Curve
STS	Society of Thoracic Surgeons
AKI	Acute Kidney Injury
AR	Augmented Reality
ICU	Intensive Care Unit
