# Supplementary material for: The Role of the Cardiothoracic Surgeon in the Age of AI—Are the Robots Going to Take Our Jobs?
Source: Med Sci (Basel). 2026 Mar 25;14(2):164. doi: 10.3390/medsci14020164 (PMC13108180; doi:10.3390/medsci14020164)
Supplement: Supplementary file 1 [file medsci-14-00164-s001.zip › medsci-4152687-supplementary/Supplementary File S1.pdf]

Complete Literature Search Strategy

The literature search aimed to identify studies evaluating artificial intelligence (AI), machine learning (ML), and robotic technologies in cardiothoracic surgery. Searches were conducted in five databases: PubMed, Scopus, Web of Science, Cochrane Library, and Google Scholar, covering publications from January 2000 to May 2025.

Search terms were developed using combinations of MeSH terms and free-text keywords, linked with Boolean operators.

Database	Search Query
**PubMed**	("artificial intelligence"[Title/Abstract] OR "machine learning"[Title/Abstract] OR "deep learning"[Title/Abstract] OR "computer vision"[Title/Abstract]) AND ("cardiothoracic surgery"[Title/Abstract] OR "cardiac surgery"[Title/Abstract] OR "thoracic surgery"[Title/Abstract] OR "coronary artery bypass"[Title/Abstract] OR "CABG"[Title/Abstract])
**Scopus**	TITLE-ABS-KEY ("artificial intelligence" OR "machine learning" OR "deep learning") AND TITLE-ABS-KEY ("cardiothoracic surgery" OR "cardiac surgery" OR "thoracic surgery" OR "robotic surgery")
**Web of Science**	TS=("artificial intelligence" OR "machine learning" OR "deep learning") AND TS=("cardiac surgery" OR "thoracic surgery" OR "cardiothoracic surgery" OR "robotic surgery")
**Cochrane Library**	("artificial intelligence" OR "machine learning") AND ("cardiac surgery" OR "thoracic surgery" OR "cardiothoracic surgery")
**Google Scholar**	"artificial intelligence" AND "cardiac surgery" OR "robotic cardiothoracic surgery" OR "machine learning cardiac surgery"
