# Supplementary material for: The Role of the Cardiothoracic Surgeon in the Age of AI—Are the Robots Going to Take Our Jobs?
Source: Med Sci (Basel). 2026 Mar 25;14(2):164. doi: 10.3390/medsci14020164 (PMC13108180; doi:10.3390/medsci14020164)
Supplement: Supplementary file 1 [file medsci-14-00164-s001.zip › medsci-4152687-supplementary/Supplementary Table S1.pdf]

Risk-of-Bias Assessment and Study Characteristics

Risk of bias was assessed using:

- Newcastle–Ottawa Scale (NOS) for observational studies
- Cochrane Risk of Bias Tool (RoB2) for randomized trials

Studies scoring ≥6 on NOS were considered moderate-to-high methodological quality.

Study	Study Type	Design	Sample Size	Assessment Tool	Score	Risk of Bias
Allyn et al.	Clinical prediction	Retrospective cohort	6,889	NOS	7/9	Low
Benedetto et al.	Systematic review/meta-analysis	Observational synthesis	22 studies	NOS adapted	8/9	Low
Penny-Dimri et al.	Registry analysis	Retrospective cohort	153,932	NOS	8/9	Low
Khalaji et al.	Clinical prediction	Retrospective cohort	16,850	NOS	7/9	Low
Fan et al.	Clinical prediction	Prospective cohort	5,443	NOS	8/9	Low
Allou et al.	AI model validation	Observational cohort	40,000+	NOS	8/9	Low
Bodenhofer et al.	Risk prediction	Retrospective cohort	2,229	NOS	7/9	Low
Lee et al.	AI prediction	Retrospective cohort	3,412	NOS	7/9	Low
Nemati et al.	ICU prediction model	Retrospective cohort	31,000	NOS	8/9	Low
Meyer et al.	ICU complication prediction	Retrospective cohort	24,000	NOS	7/9	Low
Valdis et al.	Surgical training	Randomized trial	60	Cochrane RoB2	Low	Low
Atroshchenko et al.	Simulation training	Prospective experimental	40	NOS	7/9	Low

Study	Study Type	Design	Sample Size	Assessment Tool	Score	Risk of Bias
Gong et al.	Surgical outcomes	Retrospective cohort	540	NOS	7/9	Low
Darehzereshki et al.	Surgical outcomes	Retrospective cohort	1,200	NOS	7/9	Low
Cao et al.	Meta-analysis	Observational synthesis	10 studies	NOS adapted	8/9	Low

Summary of Study Designs

Among the 67 included studies:

Study Design	Number of Studies
Retrospective observational studies	38
Prospective cohort studies	16
Randomized trials / experimental training studies	7
Systematic reviews / meta-analyses included for evidence synthesis	6

Overall methodological quality was moderate to high, with most studies demonstrating clear outcome reporting, appropriate model validation, and adequate sample sizes.
